# Interest contagion in violation-of-expectation-based false-belief tasks

**DOI:** 10.3389/fpsyg.2014.00023

**Published:** 2014-01-30

**Authors:** Andreas Falck, Ingar Brinck, Magnus Lindgren

**Affiliations:** ^1^Department of Psychology, Lund UniversityLund, Sweden; ^2^Department of Philosophy and Cognitive Science, Lund UniversityLund, Sweden

**Keywords:** interest contagion, false belief, theory of mind, memory, attention, development

## Abstract

In the debate about how to interpret Violation-of-Expectation (VoE) based false-belief experiments, it has been suggested that infants are predicting the actions of the agent based on more or less sophisticated cognitive means. We present an alternative, more parsimonious interpretation, exploring the possibility that the infants’ reactions are not governed by rational expectation but rather of memory strength due to differences in the allocation of cognitive resources earlier in the experiment. Specifically, it is argued that (1) infants’ have a tendency to find more interest in events that observed agents are attending to as opposed to unattended events (“interest contagion”), (2) the object-location configurations that result from such interesting events are remembered more strongly by the infants, and (3) the VoE contrast arises as a consequence of the difference in memory strength between more and less interesting object-location configurations. We discuss two published experiments, one which we argue that our model can explain (Kovács etal., 2010), and one which we argue cannot be readily explained by our model (Onishi and Baillargeon, 2005).

## INTRODUCTION

In recent years a number of experiments have established that infants successfully predict actions by people holding false beliefs (for a review see Baillargeon et al., 2010). Several of these false-belief tasks are based on violation-of-expectation (VoE) measures, designed to tap participants’ surprise in response to unexpected stimuli. A representative example is Onishi and Baillargeon’s (2005) seminal false-belief study that examined 15-month-old infants’ ability to predict an actor’s behavior on the basis of her true or false-belief about a toy’s hiding place. They measured the time during which the infants looked at specific events, taking looking-time as an indicator of surprise. The events were construed so that they were more or less expected from the infant’s perspective, depending on whether the infant expected an agent to act rationally according to her belief. Onishi and Baillargeon (2005) found that when the actor searched for the object where the actor had left it, the infants looked for a shorter time than when the actor searched at the location to which the object had moved in the agent’s absence. This was interpreted as the infants’ expecting the agent to act according to her belief, showing that infants at 15 months of age are sensitive to what other people believe. Onishi and Baillargeon’s (2005) study sparked a debate regarding the complexity of 15-month-olds understanding of other minds. On some accounts, the infants may indeed be predicting what the actor will do next, but basing their predictions on a weaker form of representation than that of another person’s belief. Thus, Perner and Ruffman (2005) suggest that infants form a behavior rule stating that “agents look for objects where they last saw them,” and Apperly and Butterfill (2009) suggest that infants approximate beliefs based on perceptual access. On other accounts, the infants are not making predictions based on past events, but their looking behavior is instead governed by associations either between agents, objects, and locations (Perner and Ruffman, 2005) or between locations, objects, and object affordances (Bruin et al., 2011). However, it may not be optimal to search for one single mechanism in order to explain infants’ behaviors in these tasks. Many different mechanisms may be at play concurrently in a specific situation, and over the course of development they may interact and build on each other.

**FIGURE 1:**
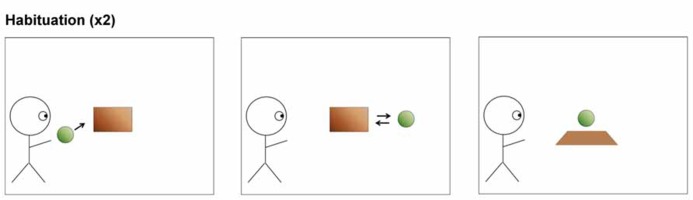
**Infants were habituated to a movie with the depicted structure.** The ball is thrown so that it passes and then hides behind the occluding wall and is subsequently revealed, all in the agent’s presence. Each infant was shown this animation twice (Kovács et al., 2010, supporting online material).

**FIGURE 2:**
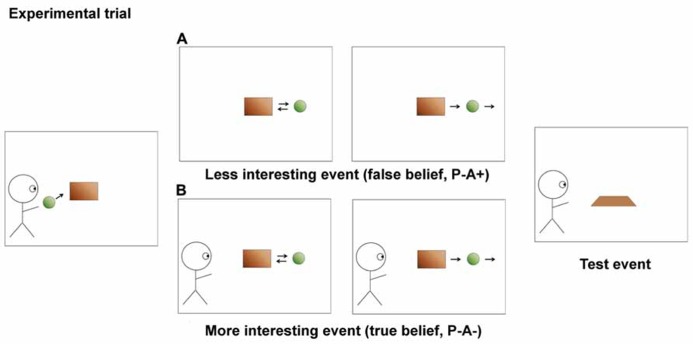
**The two experimental conditions from Kovács et al. (2010; experiment 5)**. In condition **(A)** the agent’s and the infant’s beliefs differ, but this also renders the depicted event less interesting to the infant, compared to condition **(B)**. When the occluder is lowered in the last panel, the infants in condition **(B)** will have a stronger memory of the events, leading to shorter looking time compared to the infants in condition **(A)**. The condition names in parentheses are from Kovács et al. (2010) paper.

Kovács et al. (2010) employed a paradigm similar to the one employed by Onishi and Baillargeon (2005), in order to show that 7-month-old infants were sensitive to situations to which an agent previously had attended. Infants were presented with animated movies in which an agent (a blue smurf) was watching a ball rolling in and out behind a barrier. After two habituation trials (**Figure 1**), in which the ball ended up behind the occluding barrier and subsequently was revealed, each infant was presented with one of two experimental movies (**Figure 2**). In both movies the ball left the scene at the end; the conditions differed as to when the agent left the scene. In one condition, the agent had seen the ball roll off the scene, in the other condition the agent had last seen the ball roll in behind the barrier. Then, the barrier was lowered and revealed no ball, which was consistent with the experimental trial in which the ball had disappeared in both of the conditions. However, from the habituation trials the infants were nevertheless accustomed to seeing the ball behind the occluder. The infants who had seen the ball disappear in the agent’s absence (**Figure 2A**), such that the agent would still “believe” the ball to be there, looked longer than the infants who had seen the ball disappear in the agent’s presence (**Figure 2B**). This suggests, according to Kovács et al. (2010) that the agent’s apparent false-belief that the ball would still be present behind the occluder influenced the infant’s own belief about the ball. Their experiment raises an important question about what the looking-time response indicates. Does it show what the infant expects the agent to do, or does it merely show that the infant is sensitive to specific object-location configurations as opposed to others?

A few studies have manipulated the agent’s presence independently of the agent’s looking behavior, effectively ruling out a simple association model based only on the presence of the agent, at 13 months of age (Southgate et al., 2007) and at 18 months (Senju et al., 2011). However, seeing implies another aspect that so far has not been controlled for. We submit that in most infant false-belief experiments, the fact that the agent perceives or attends to a certain event increases the infant’s interest in that event, so that the infant finds events attended to by others more interesting. We dub this mechanism “interest contagion”, because we hypothesize that (a) attention is a signal of interest, (b) what others show interest in will also seem interesting to an observer, and (c) the transfer of interest is involuntary or mandatory and thus may be properly viewed as contagion. We will argue that events found interesting by the infant give rise to stronger memory of these events, something that in turn causes differences in looking time responses in false-belief experiments. For instance, a hiding event that the agent does not see is perceived as less interesting, leaving the infant with a weaker memory of the situation in which the object is at the hidden location, than does an event that the agent sees. This difference in the infant’s memory of specific situations is sufficient to create the difference in looking time in the experiment by Kovács et al. (2010). We will articulate our argument with respect to that experiment, claiming that interest contagion can explain the data, and then discuss whether interest contagion can inform the interpretation of other experiments such as that of Onishi and Baillargeon (2005).

## INTEREST CONTAGION IN FALSE BELIEF TASKS

By “interest contagion” we mean the tendency to find interest in what others attend to. Similarly to how “emotion contagion” is defined by Hatfield et al. (1994) as “The tendency to automatically mimic and synchronize expressions, vocalizations, postures, and movements with those of another person’s and, consequently, to converge emotionally,” we define interest contagion as follows:

The tendency to automatically find interest in events, situations, or objects that others show interest in, as conveyed by behaviorally manifest attention, and, consequently, to converge with respect to interest.

Extensive research on social aspects of attention (for a review see Birmingham and Kingstone, 2009) has shown that we tend to look where other people look. Already infants as young as 6 months follow the gaze of others (Reid and Striano, 2005). ERP results from 4-month-old infants suggest that objects upon which an adult had previously gazed are detected as more familiar by the infants, compared to objects that were not gazed at (Reid et al., 2004). Further effects in the same vein have been shown in the context of joint attention, both in 9-month-olds (Kopp and Lindenberger, 2011) and in 4-month-olds (Kopp and Lindenberger, 2012). Interest contagion need not be contingent on overt gaze: head direction is cueing attention independently of gaze in a way that leads to slowing of reaction times in adults when concurrent head and gaze cues mismatch (Langton, 2000). One-year-olds find interest in otherwise uninteresting objects when adults act on them (Trevarthen and Hubley, 1978), suggesting that interest is conveyed by a range of goal-directed behaviors when the agent’s attention to the goal is manifest.

Hence, interest contagion is a consequence of agents’ bodily manifest attention. What agents attend to or do not attend to (or see) is exactly what is modulated in the false-belief experiments. These experiments are built up as narratives with few distractors other than one or two agents and an object which subsequently is moved around. Typically, the critical false-belief contrast is created by letting an agent see some events involving the object while failing to see other events.^1^ The critical location change in the false-belief condition goes unnoticed by the agent but the agent sees the location change in the true-belief condition. Our hypothesis is that the memory of the object at the final location will be stronger for an infant facing the true-belief condition than for an infant experiencing the false-belief condition. A stronger memory of a situation would render the situation less surprising to the infant, shortening the time during which the infant is inclined to look in response to subsequent presentations of the same situation. Thus, the difference in looking-time between the conditions can be explained as a result of the infant’s sensitivity to other agents’ attention. Hence, there is no need to assume that the infant is viewing the agent as rational, acting on his or her beliefs.

An agent’s action can be surprising if it is not rationally justified by the perceived events, for example when an agent searches for an object in a place where it is irrational for her to search. However, surprise can also be an effect of pure frequency, since an infrequent stimulus comes out as surprising in comparison with more frequent stimuli. If the agent were to repeatedly interact with an object at one of two locations, and then suddenly turn to the other location, the new interaction would be more surprising. This contrast is employed in habituation experiments such as those of Onishi and Baillargeon (2005) and Kovács et al. (2010, experiments 4–7). Infants are habituated by being shown repeated instances of a specific event (i.e., an object hiding at a specific location), before being shown more or less expectable situations based on the same pattern. For example, in two of Kovács et al.’s experiments (2010, experiment 5 and 7), at first the ball is repeatedly shown ending up behind the occluder and revealed when the occluder is lowered. Then it is shown leaving the scene in a subsequent experimental trial. In the test phaseduring whichthe infant’s looking time is measured, the occluder is lowered and shows an empty space, which corresponds to what you would expect having seen the ball leave, but not to the previously habituated state. This relative familiarity of the habituated situation compared to the test situation is assumed to affect the looking time, as the habituation logic behind the experiment makes evident.

However, the mechanism behind the habituation is simply that subsequent presentations of a specific situation strengthen the perceiver’s memory of that situation. If different stimulus configurations were equally common, the perceiver’s interest when viewing the events would affect which stimulus would leave the stronger memory, and consequently, which stimulus would be the more surprising at subsequent presentations.

## INTEREST CONTAGION APPLIED TO EXISTING EXPERIMENTAL PARADIGMS

In Kovács et al.’s (2010) experiment number 5, all events were equal in the two conditions, except for that the agent left at different points in time in the different conditions. We explain the results of the experiment as follows. In the critical false-belief condition, the agent left before the ball rolled out from behind the occluder and rolled off the scene. That the agent left at that point rendered the agent’s belief false, and also made the ball-leaving event less interesting from the perspective of the infant. In the corresponding true-belief condition, the infant sees the agent watch the ball roll off the scene, rendering the ball-leaving event more interesting from the infant’s perspective. Since interesting events cause stronger memories, the infants who saw the agent watch the ball leave (the true-belief condition) were left with a stronger memory of the ball’s leaving than infants who witnessed the false-belief condition. Thus we expect that the looking time in the test situation, in which the occluder is lowered revealing no ball, will be shorter for the infants in the true-belief condition than for the infants in the false-belief condition. This is precisely what is found by Kovács et al. (2010, experiment 5).

It is an open question if 7-month-old infants make predictions based on representations of other peoples’ beliefs, rather than merely reacting to their behavior without attributing a rational structure to the events. Parsimony speaks in favor of our interpretation of Kovács et al. (2010) experiment. Drawing on recognition memory as the crucial cognitive mechanism, we avoid making the assumption that infants’ predict what is going to happen next, based on representations of past events. This distinguishes our account from all forms of perceptual access accounts, whether they build upon representing other’s beliefs (Baillargeon et al., 2010), approximations of beliefs (Apperly and Butterfill, 2009), or what others previously have seen (Perner and Ruffman, 2005). These accounts have in common that they suggest that infants base their predictions on what is rational for the agent to do given his or her past experience. Our account predicts that the infant will not be sensitive to the presence of the agent in the test phase, since the facilitatory effect of the agent occurs only in the phase in which the to-be-tested association is formed. Indeed, this is what Kovács et al. (2010) find in an adaptation of the described experiment, both in adults (experiment 2) and in infants (experiment 7). Moreover, this is not predicted by an association-based account along the lines of Perner and Ruffman (2005), or by the affordance-based account by Bruin et al. (2011). Since Kovács et al. (2010) find their effect regardless of whether the agent is present in the test phase or not, clearly, their experiment does not provide evidence that the participants’ reactions to the test event is dependent on the context as given by the agent’s presence.

However, turning to Onishi and Baillargeon’s (2005) original experiment, interest contagion alone cannot explain the infants’ looking behavior. In contrast to Kovács et al. (2010), Onishi and Baillargeon (2005) used two hiding locations, which enabled them to compare looking times to each location directly in the experimental conditions. Thus, they were able to show that in some of their conditions, the habituated location produced longer looking times than the other location. Still they consistently found that the infants looked less to searches in the locations where the agent last saw or interacted with the object. Taken together, this suggests that at 15 months, at the very least, infants (1) have a robust sense of when the state of the world has changed, and so can disregard the habituated location when the object has moved, and (2) upon the agent’s return are able to disregard information from situations other than the one associated with the agent. From our perspective, an intriguing possibility is that what drives the infants’ behavior also in this case is not the mere presence of the agent, but rather the overt interest shown by the agent. Thus, only an interested agent will be associated with the relevant situations and events memorized by the infant. This would also be compatible with the results by Southgate et al. (2007) and Senju et al. (2011), in which the agent never left the scene but failed to see the critical event.

## OUTSTANDING QUESTIONS

Finding decisive evidence in favor of one account of infant social abilities over another has proven hard, mainly because of the inherent confound of agents’ seeing, knowing and showing overt (manifest) interest present in the experiments. Even if an experiment would show that a non-social cue gives rise to the same looking time pattern as a social cue, it would not be easy to show that the mechanism was one and the same in both cases. In any case, interest contagion is worth exploring in its own right. We can identify at least three areas of inquiry emerging from the hypothesis suggested here.

• Which aspects of an agent’s behavior convey interest robustly?
• Which types of agent-location configurations provide contexts for remembering situations?
• When and how do infants become sensitive to such contexts, in addition to being susceptible to interest contagion?

These questions can be studied by parametrically varying different aspects of an agent’s overt interest independently of the agent’s seeing, in experiments with a structure similar to the false-belief tasks of Kovács et al. (2010) and Onishi and Baillargeon (2005), respectively. Thus, the first question can be evaluated by manipulating the agent’s behavior in the first phases of the experiment. We predict that doing so would render the events leading up to the final location of the object more or less interesting to the observer, creating differences in looking times or reaction times. When such a paradigm is established, the agent’s behavior in the test phase can be manipulated in order to explore under which circumstances the context given by that behavior plays a role for the observer’s reactions, addressing the second question. Finally, we may employ the outlined paradigm with infants at different ages, framing how sensitivity to the contextual effectsof other agents’ behavior develops over time.

## CONCLUDING REMARKS

We suggest that interest contagion as spelled out here may be a constitutive part of an observer’s appreciation of other people’s perception. As such, it is not a mere confounding factor due to shortcomings of experimental design. Furthermore, the cognitive effects of being interested in what others attend to or show interest in may produce effects on infants’ looking times similar to those found in VoE based false-belief experiments. The mechanism proposed here accounts for the results of Kovács et al.’s (2010) false-belief experiment with 7-month-old infants. While not explaining results from older infants, it suggests directions for how to look for similar mechanisms for other VoE results, such as those from Onishi and Baillargeon (2005). Our account of early sensitivity to the perceptual history of other agents is minimal and does not presuppose that infants construct a rational interpretation of the perceived events. The underlying mechanism can clearly act as a supporting mechanism in social cognition and the understanding of social situations, as well as developmental scaffolding for more elaborate sociocognitive abilities.

## Conflict of Interest Statement

The authors declare that the research was conducted in the absence of any commercial or financial relationships that could be construed as a potential conflict of interest.

## Footnotes

1 An exception from this observation is Song et al. (2008), in which the agent was told about the location of the object.
